# Naval Hygiene

**Published:** 1853-08

**Authors:** G. R. B. Horner

**Affiliations:** Philadelphia, Surgeon U. S. Navy


### MEDICAL EXAMINER,

AND

### RECORD OF MEDICAL SCIENCE.

NEW SERIES.—NO. CIV.—AUGUST, 1853.

### ORIGINAL COMMUNICATIONS.

*Naval Hygiene.* By G. R. B. Horner, M. D., Philadelphia, Surgeon U. S. Navy.

This branch embraces a great variety of subjects relating to the health of seamen. On it their strength, spirit and, necessarily, their efficiency depend; without it commerce must suffer, and in time of war the honor and defence of the country could not be maintained against a powerful maritime nation, whose seamen are as brave and numerous and more healthy than our own. It becomes us, then, to consider the means best adapted to prevent this, and, if possible, to render them superior in every respect. At the same time we do that, we must not neglect to refer to the means suitable to preserve the health of our extensive mercantile marine. For these purposes the first means to be taken are those intended to procure vessels properly constructed regarding the comfort and health of their occupants; for, if they be confined to small, filthy and ill ventilated apartments, other means taken may be of no use. Ships of war are often built for sailing and fighting only, merchant ships for beauty and holding large cargoes.

Hence, the quarters, in both, for the crews, and especially for the men and officers of subordinate grades, are very small proportionally to their number, besides being so constructed that pure air is nearly unknown in them. It is even common to witness this in vessels wherein the cabins and saloons, intended for the passengers and officers of the highest grades, are as spacious and airy as they can be made. In no class of vessels are we more struck with these facts than in packets and steamers. In the latter, engaged in trade between the eastern coast of the United States and the western coast of California, I observed this contrast in accommodations. While the cabin and steerage passengers were lodged mostly in separate state rooms and had sumptuously furnished saloons or cabins adjoining, the forward passengers were crowded in berths near the bows or about the forecastle. Some of them were occupied by two or three persons in each one, and were so short that no man of good height could lie in them at full length. To escape from such discomfort, which was rendered intolerable from the heat of the tropical sun added to that issuing from vast fires of coal, many passengers fled nightly to the highest decks and slept upon them. Of the sufferings of the forward passengers we may form an estimate, when informed that a number of the best accommodated cabin ones deserted their rooms and slept on the quarter deck beneath the awnings, so great was their heat. To ship builders and naval engineers belong the general construction and arrangement of vessels, and we would not recommend any medical interference with them in most matters, or where mere comfort and convenience are concerned. But in things relating to health, courtesy as well as public and private interests demand that medical men should be consulted. Whether these be or not, the former should endeavor at least to have all parts of a ship made salubrious; to have the hatches well placed for both air, light and the escape of effluvia; the air ports of large size, and so situated that they could be opened either in calm or stormy weather without the admission of rain or sea water. For the escape of heated air, animal and other exhalations, the newly adapted ventilators passing through the upper decks in parts not used as gang ways and not occupied by guns, &c., ought to be preferred. These ventilators have tops closed above and open at the sides, and answer the indications above mentioned, and from being of cast iron are very durable.

To ventilate perfectly and expel noxious gases, as well as overheated air, windsails are indispensable. They are very large and long cylinders or bags of canvas, open at bottom and expanded at top into a capacious mouth, presented to the wind, and held open by lines attached to their elongated sides. Hoops within prevent their collapse. These windsails are suspended from the rigging by other lines, and can be let down through the hatches far enough to ventilate the lowest parts of vessels. All those of our navy are thus purified, and made vastly more healthy and comfortable than ships were before the introduction of windsails.

But the part of every vessel most demanding the care of the physician is that used by the infirm, and known in men-of-war as "the sick bay." In those of our country it is almost without exception on the berth deck, either in the bows or amidships between the fore and main hatch. The former place is its location in ships of the line, and frequently the latter is that in vessels of smaller size. In our steamers the machinery will not allow of the sick being kept amidships, and abaft there is no room for them; hence from necessity they have to remain forward. This is the case even in the San Jacinto, one of our largest steam frigates, and more than 220 feet long. She indeed has no separate place for the sick; they have to lie near the fore hatch of the berth deck, and are exposed to both the heat of the galley or cooking range just abaft them, and to the intense fires in the adjacent furnaces generating steam, in the hold below. She has no gun deck nor other place of refuge (save the spar one) from such heat, for the halt or sick, by day or by night; and if it rain, or the weather be cold, or the wind strong, they have the unhappy alternative of bearing its chilling blasts, or of closing the hatches and being stewed in steam. From the description by the crew of what they suffered from these causes—the flooding of the berth deck by the sea through the horse holes at the bows of the same deck, the deluging of it by rain, the seas shipped, and the water thrown upon it in the coldest weather to clean it—we can rationally infer, that though she cannot be called a floating hell, she might be well termed a steam purgatory. Of the Saranac the same remark might be applied with good reason.

In the English ships of war I have visited, the sick were placed on one of the gun decks, and on both or only on one side of the bowsprit. This position is most desirable from its being generally the least in the way; but when the men are letting out or heaving in a cable, or the ship is in a heavy sea, plunging deeply and buffeted by waves driven on by a head wind, it is very noisy, and often deluged by the water forced into the ports, seams and horseholes, or those through which the cables pass. The motion, too, in the bows of a vessel, is very great at such times, and renders them altogether unfit for patients ill of low fevers, from the depressing effects on the brain; or for those who suffer much from sea sickness, or who have fractured limbs. In these cases, while ships are at sea, it is always necessary to remove the patients abaft, and I usually place them near the middle of one of the gun decks. While I was in the John Adams, on a voyage from Minorca to Naples, thence to Marseilles, and back to the former port, a most striking instance occurred of the ill effects of a ship's motion on a case of the first kind, or that of low fever. The patient was a boy of that island, for the first time at sea, who suffered from excessive sea sickness and intermittent fever, to which he was liable. Accordingly, as the wind was light or strong, the sea smooth or rough, the ship in port or out of it, easy or uneasy, so the type of the fever was of ordinary height or of the lowest kind, and deprived him of nearly every sign of animation. When thus affected, his teeth were covered with dark sordes, his eyes sank, his pulse was rapid and scarcely perceptible, his face livid, and he sank into such a state of insensibility, that he was only to be kept alive by quinine, applications of turpentine and cantharides ointment, and other stimulating remedies. In this manner he lived until he got back to his native place; but when she was about returning to Naples, he could not remain in her, was sent to the Lazaretto of Port Mahon, and there died under the charge of a Spanish physician. Reference has been made to this case in my work on the Mediterranean; but it has been again introduced, as it is finely illustrative of the bad influence of a ship's motion on some patients.

To make the sick more comfortable, it is also very essential that they have a privy convenient to them, and be as little as possible exposed to being struck by the sun; that they should have well made and covered easy chairs with copper pans, which are difficult of being broken, and require a long time to become corroded. Porcelain pans are too easily fractured, and tin ones very soon are rusted and rendered useless. A hatch directly over a sick bay is of great advantage; it affords plenty of light, admits a windsail, and enables the medical officers and patients—both those affected with disease and those injured by accident—a ready way of egress and ingress. When there is no such hatch, and a person is severely injured, it becomes necessary to drag him during sleeping hours beneath the hammocks of the crew to get him into the hospital. To do this is difficult and painful to him and carriers, from the hammocks being hung only a few feet above the deck, and obliging the latter to stoop very low while they drag the former. Another great convenience is that of having the dispensary and store room adjoining or near the sick bay, so that articles can be had for patients without delay. But in time of war the greater part of medical stores should be below water mark, and secure from damage by an enemy's shot. A single one, and especially an explosive shot, might otherwise smash all the bottles of the medical department and leave no liquids for use. This misfortune might frequently happen in our men of war of the present time, for in all of the corvettes, and in most if not all our frigates, the dispensary is on the berth deck. In former times it was in the cockpit of the last, and below water mark. Want of air and light, and the inconvenient distance of the former from the sick, have probably caused the change of place, besides many annoyances from the numerous store rooms opening into the cockpit, and its proximity to the spirit room and after magazine. When these are open no lights are allowed to be burnt, and of course the darkness prevents the compounding of medicines and other work being done. When they are immediately required, this is a great inconvenience to the sick and their attendants. In time of action, or any other, when the crew are at quarters, the wounded or injured sent to the cockpit must receive attention, and the use of covered lights is then permitted.

With a hospital arranged as above described, patients, unless over-crowded or affected with contagious diseases, will mostly improve under judicious medical advice. Should any be affected in the last manner, they ought to be forthwith sent on shore where it be practicable, or placed in the most airy and least frequented parts of the decks, either lower or upper. There they should be hung in cots; or hammocks, if there be none of the former to be had; put behind screens, and allowed to hold no communication with any other persons than their nurses and physicians. If small-pox exist it will be very desirable that the former should have had it by inoculation or naturally, or that they be known to be well protected by vaccination. But, should this sovereign preventive have been properly practised among a crew before or after shipment, it is very improbable that they will ever become extensively infected with small-pox. Therefore, every one of a crew in the least liable to it should be vaccinated as soon as he gets on board ship, and while the vaccine matter can certainly be obtained fresh. If it be not, it will prove useless, according to my experience;—for, of the many hundred persons I have vaccinated or caused to be, in various ships of war at sea, scarcely one gave any evidence of having been infected with the virus. The same remark was made by a surgeon of much greater experience than myself. So many failures may have been owing to other causes, as gross diet—the adult state of a great majority of the crews—and their greater unsusceptibility to impression from the hardness of their skins, and loss of sensibility. Some may ascribe the failures to the influence of sea air, but none of these things are known to be facts. This, however, is an acknowledged and well proved one. Old vaccine matter has often failed to produce its peculiar effects on persons ashore with every adjuvant required to insure success. Neither the purest air, nor best diet, nor well adapted clothing and most able medical advice have been of any service. The punctures have, notwithstanding, healed and left no cicatrices, and the vaccinated have remained unprotected from variolous contagion. To escape it in very cold weather is most difficult, from it being necessary then to make vessels of comfortable temperature; from their crews being thickly clad, and seldom changing their clothes; staying much below decks; crowding together in warm places; closing ports and hatches, and stopping ventilation. When this cannot be done, they become exposed to too much cold, suffer from pulmonic complaints, rheumatism and chilblains, and hence have a choice of the two evils—contagion, or the suffering caused by these affections. Sometimes a ship may be kept very close below and open above, as was the Brandywine in the winter of 1830, at New York. To that fault we ascribe the complication of the above diseases herein suffered. In her were mingled cases of small pox, scarlatina, typhus fever, and every other affection it seemed which could be induced by intense cold. Luckily some of the former cases were sent to the naval hospital before the crew were extensively infected. But by the time the ship left port, in March, she had lost one man from scarlatina, sent thirty altogether to the hospital, and had forty more on the sick list. In a few days after sailing, three more persons died of the same disease and typhus, and in the course of a few months others of the crew had been similarly lost. Among them was the sergeant of marines, Clargis, a noble looking, accomplished soldier, who had had a violent pneumonia terminating in mortification of the lungs. His sufferings were indescribable; so great and protracted were the dyspnœa and difficulty in expectorating the exceedingly fœtid sputa which for day after day were discharged, to the disgust of the whole crew.

So much was due to the fitting out of a man of war in winter! a practice ever to be condemned or avoided. There are two other practices in ships almost as condemnable; one is, that of dry *holy stoning*, or rubbing the decks with huge stones and sand; the other practice is that of deluging the former with water in the coldest weather, even when it freezes as soon as thrown upon them. Dry holy stoning is intended solely for good appearance and cleanliness, washing decks for the same purposes and to keep them tight. In doing this the men generally go barefooted, and suffer proportionally from the conjoined ill effects of cold and moisture, inducing rheumatic and pulmonic complaints. By holy stoning the chief mischief is done to the eyes and lungs, which inhale the impalpable sand floating in the air, and become irritated. To this cause is attributed the death of the master-at-arms belonging to the vessel named, and who became affected with phthisis pulmonalis. Cases of ophthalmia arise and are aggravated from the same cause. But when the sand is wet it may be rubbed on the deck without injury; and in mild weather washing them does little harm—unless on the lower ones—from their continuing wet a long time, and may be of advantage, from the men getting their feet cleansed, who, if the decks were not washed, would seldom have them so. To obtain a like condition of the whole person, bathing in fresh or sea water should be practised by a crew, whenever it be of moderate temperature. Though buckets and tubs sufficient could not be had for a large number of men, they might bathe on a beach, or at sea, by having boats rowing about them and letting down sails into the water by the four corners for the use of inexpert swimmers, as I have often witnessed, when the sea has been calm. Bathing, besides being a very healthy practice, from its tonic and cleansing effects, is a fine amusement, and for this reason, if no other, ought to be encouraged. Like music, dancing, playing at dice, chess, draughts and dominoes, it takes away the monotony of sea life, makes time pass pleasantly, and creates good will in a crew. Water is still more highly recommended for drinking, and unless it be pure, it is vain to expect good health in any vessel on a voyage, and cut off from the means of procuring substitutes when it is impure. When good, it is the only drink seamen can use freely with general impunity ; and nothing laid in for their sustenance makes them so efficient and so comfortable. It is an indispensable article with them, and one for which no substitute can be found. This is not so with any other ; if the pork be bad, the beef may be good; should the bread be mouldy and wormy, the rice may be sound; if the cheese be spoiled, an additional quantity of butter can be allowed. The same may be done with any other part of the rations. Bad water, too, may be as injurious in port, and sometimes more so than at sea, when sailors at the same time indulge in eating fresh meat, fruits and vegetables, especially the crude, unripe and acid ones. If these be eaten at the same time that brackish water is drank, or that obtained from fountains and rivulets just after heavy rains, and charged with earthy matter, they never fail to produce bowel affections. The greatest number which have ever come under my notice have been caused in this manner; and I always expect as a matter of course, when between the tropics, or it be summer anywhere, to have at once a large number of such affections to treat. But by far the largest number have been seen among crews drinking the water of the La Plata, or that taken from its shores. Neither should it be used before it is purified by being allowed to work, or by strainage, or throwing into each cask a small amount of powdered alum, which is thought at Buenos Ayres the best purifier, and ought to be proportioned to the quantity and impurity of the water. For the consumption of a small number of persons, dripstones may furnish as much pure water as they may need for drink; and in the Mediterranean, from the vast quantity of porous sandy limestone found on its shores and islands, these stones can be easily obtained at a low price. At Malta they are very plentiful, and so cheap, that every vessel touching there ought to lay in a supply for both present and future use; for after they have been used for some time, the impurities of the water strained fill their pores and impede its flow, so much as to prevent this being abundant. Should a crew be large, other means must be adopted, and none answer so well as large casks, set on end like a common scuttle-bat, and having either a strainer at bottom made of several layers of bunting or of hair cloth, resting on a grate and covered with gravel and sand; or having a patent strainer of silver gauze, with a clean sponge fixed inside of it. The frigate Savannah had a strainer of the latter kind, which was attached to the scuttle-bat when necessary, and was thought very good. For the use of my patients in the Delaware I employed a strainer of the former kind, holding only five or ten gallons of water, and for the same purpose in the Savannah used a large stone filter, which had a double bottom and a chamber above, with a hole in the centre into which a sponge was placed. The water ran through this, thence percolated through a layer of sand and a strainer beneath—got into the lowest chamber and was drawn off by a stop-cock in a limpid, pure condition. For a sick bay I prefer this filter* to any other, from its convenient size, easiness of being cleansed and repaired, and so well answering the purpose for which it is designed. Other filters perhaps may be obtained to answer as well, and there is no excuse for a vessel leaving, at least any of our ports, without having some of them. In steamers there is less demand for them, from the great ease with which their immense fires can by means of retorts or alem-

*It is said to be one of Wainwright's of Staffordshire, England, which have gravel and charcoal mixed with the sand, contained in the middle or closed chamber.

bics, convert salt water into fresh. Whether water thus distilled is as wholesome as that impregnated with some saline or earthy matter, is a subject of doubt, as in some persons it has caused diarrhœa, and it, moreover, when so purified, is insipid and unpalatable.

Of the best methods of preserving and purifying water, much has been written. Some have recommended a small amount of quick lime and sulphuric acid to be put into each cask; some have used muriatic acid, others calcined lime or pure charcoal, with a little sulphuric acid; others have put in powdered charcoal alone, or charred the casks inside. This is a very common method of purifying them when old, and though it may not have any great efficacy in preserving the water, it certainly has some in preventing the decay of the casks. The charcoal itself, likewise, when put into the water, acts both as an antiseptic and destroyer of fœtor arising from the formation of sulphuretted hydrogen. But I prefer filtrating impure water through charcoal, when confined in a chamber or other place suited to hold it and prevent its rising to the surface, as its lightness will certainly cause it to do when thrown loose into the water.

*Provisions.*—Such a variety of them are now used at sea, that it would be superfluous to enumerate them or do more than write a few remarks respecting some of the principal. Of these the two most used are bread and meat. The former in all ships is commonly biscuit made of wheat flour,—sometimes of this and rice mixed, when the former is very dear, as in Brazil. The hardest bread I ever saw was obtained at Bahia, and was said to have been thus made. It was too hard for any ordinary teeth to chew, and required hammering to be got into fragments. It is hardly necessary to say that such bread, and the wormy, mouldy and sour, should be condemned. Indeed biscuit generally is difficult of mastication and digestion, from its toughness, and is neither savory nor wholesome, unless dried and crisped in an oven just before it is eaten. Next to bread, rice may be ranked as the most valuable vegetable food. Though it is insipid, and constipates most persons, it can be easily rendered palatable by seasoning, and prevented from producing costiveness by mingling it with stewed fruit or molasses. For the sick

I have found rice invaluable in bowel affections, either in the form of a gruel or drink, or when simply boiled and flavored with any pleasant condiment. In this state my patients, almost without exception, have preferred it to arrow root or any other of the farinaceæ, which have a flatness of taste—evils to be overcome only by high seasoning with wine, sugar, lemon juice and spices, rendering them as rich as prohibited meats. Of these, any which are preserved by being first cooked—by boiling or roasting—are to be preferred for the sick or well, to all dried ones, or any preserved by being put into brine, either of salt alone or that and nitre. Both of these articles so impregnate flesh of all kinds as to make it hard and insoluble in the gastric juice. It then creates irritation of the stomach, causes unnatural thirst, and is altogether difficult of assimilation, besides impregnating the system with a superabundance of saline matter. The first ill effects observed afterwards are ulcers in the mouth, phlegmous in different parts of the body and limbs, and finally scorbutic symptoms. To avoid, then, these and other evils resulting from salt provisions, as little of them as possible ought to be used, and when they are, they should not be cooked before they have been first soaked at least twenty-four hours in fresh water, and subsequently washed in it after it has been changed. Preserved fresh meats are dear in appearance, but if we calculate their superior niceness and wholesomeness; their freedom from bone, gristle and other useless parts; their not requiring more fire than enough to rewarm them, and the very little space occupied by the canisters containing them; the very perfect manner in which these meats are kept sound for an indefinite time, and the vast amount of salt meats lost from corrosion by the brine, and putrefaction from various causes, we may very safely conclude that the latter meats are dearer than the fresh preserved. On the coast of Brazil, where these cost 45 cts. per pound, I found them decidedly cheaper than live fowls at six dollars per dozen, from the great mortality among them at sea. Even when they do not die, they often become sickly, waste away, and afford poor nourishment. The same may be said of other live stock, save, perhaps, pigs; and when not correctly, it is in rare instances,—from fine weather and great care in feeding. When this is not done, they are illy watered—the vessel labors much, tosses them about and keeps them wet; they soon droop, get feverish, and become unfit for use by the sick or well.

With fresh preserved meats we must likewise recommend vegetables which have been cooked in a similar manner and hermetically sealed in tin cases and bottles. There are also a large quantity of vegetables now reduced by heat to a very dry state and sealed in tin boxes, but when taken out and put into boiling water, resume their wonted freshness and plumpness. The best I have seen were prepared in France, and had been sent to the Chief of the Bureau of Provisions and Clothing of the Navy, for inspection and trial. They gave such satisfaction that we hope they will come into general use and be prepared on a large scale in our own country. In addition to the above articles, preserved soups, custards and milk ought to be laid up for sea stores, at least for short voyages, and as is now practised in some packets. By the aid of ice it may be easily effected, though the articles be not put in sealed vessels. In ships sailing from countries where ice abounds, it might be stored away at a very low price, in sufficient quantities to last for months, without any great inconvenience as regards room. On my passage in the Crescent City from Chagres, all our meats were said to have been preserved on ice laid in at New York, abundantly enough to last both during her outward and homeward passage. Of spirits, wines and malt liquors, it is necessary to procure a supply for culinary and medicinal purposes. Habit, too, renders them requisite for some persons, and the palates of others may make them indispensable as articles of luxury. But for the treatment of invalids, I have by long experience found them of minor importance with respect to articles decidedly medicinal. As diffusible stimulants in cases of emergency, they are serviceable; but as tonics or medicines, used to produce a permanently invigorating effect, they are much inferior to the vegetable bitters, and if any stimulation be required, it is much better to infuse these in a small amount of spirits mingled with water. This also is preferable, because it prevents patients from having it in their power to say that the "doctor recommended them to drink spirits," and to urge this as a plea for their continuance when well.

The next subject claiming our notice, and the most important to the sick, is the laying up of all the medicines, implements and appliances needed for them. In their selection we must determine how many are needed, and next the quantity of each one which may probably be required. In determining this we must be governed by the space allowed to accommodate them, the number of the crew, the length of the proposed voyage, and the amount of sickness anticipated. The ability or inability to procure a re-supply where the vessel is bound, and the abundance, cheapness or dearness of certain medicines there, ought also to regulate the quantity to be primarily obtained. It is very bad economy to fail getting a full supply of articles which are exorbitantly high abroad, and equally bad to lay in such at home when they are perhaps native products of the countries to be visited and correspondingly cheap in them. This is a familiar blunder, and committed in many vessels; and the only good reason for it is that it encourages domestic commerce and causes money to be expended at home. While laying in medical stores also, we ought as much as possible to obtain the most efficient of every class—to get them in the most concentrated form, so as to occupy the least space—to put aside crude articles and procure their active principles—and to have all medical stores, whether in drawers or boxes, well secured against rats, mice and vermin, by means of tin or other metals, and to have these well painted externally, to prevent corrosion by rust. The medicines likewise should be put in packages and bottles, perfectly air and water proof, and adapted in shape to the places wherein they are to be kept. As a general rule, the former store most conveniently and take up least space when square. It will be found in fitting up either a medicine chest or a dispensary, that when round they require about one-half more room—a vast deal to lose when not enough is allowed, although storage is made in the most economical method. One of the strongest objections to laying up an overplus of medical stores, or not packing them closely in a dispensary or chest, is that it makes it necessary to put the surplus in the hold. When this happens, they are apt to be lost and pillaged; they cannot be got for immediate use without inconvenience; and give so much trouble to those having charge of the hold that they complain, and quarrels result between them and the persons belonging to the medical department. From medicines being kept in the hold I have frequently, under the belief that there were none of a certain kind in it, obtained a fresh supply, when an abundance was there, as proved by their having been subsequently found. On one occasion I was positively informed that no alcohol and Epsom salts were in store; á re-supply was ordered and purchased, and after it had been brought on board, four gallons of the first article and a barrel of the other were discovered in that part of the hold called the spirit room. The prohibition of lights in it makes such oversights more easy; but in the latter instance it was attributed to the inscription on the head of the barrel having been covered with whitewash.

In procuring medical stores, we should likewise take into consideration the climate wherein a vessel will have to cruise or remain longest. Accordingly, if the climate is to be a cold one, we ought to lay in a full supply of all articles adapted to the treatment of rheumatism, pneumonia and other affections caused by cold. If the climate is to be a hot one, we should get an extra supply of mercurials, opium, saline compounds, acids and other remedies suited for miasmatic fevers, dysentery, diarrhœa, cholera and affections incidental to warm latitudes. In temperate climates, when we are to have an alternation of heat and cold, as in our own, we must of course obtain a supply of medical stores adapted to both. The same ought to be done in regard to clothing, and seamen of every class should be provided with such as may enable them to be well protected in any change of weather. From a want of information concerning the dampness and chilliness of that on the coast of California, it must have happened that a large amount of summer clothing was sent to our squadron there, instead of winter clothes, and occasioned great discomfort to the seamen during my stay on that station. For hot weather linen is most comfortable, but cotton is preferable, from its medium quality between the former and woolen goods, as well as for the much greater quantity to be bought for the same money. Cotton, also, does not, like linen, allow perspiration to be suddenly checked, nor does cotton so absorb perspiration and remain sodden with water. Hence muslin sheets are infinitely superior to linen for sea use. For overcoats, in frigid regions, woollen cloth is indispensable, but in rain, gum-elastic and oiled are preferable, from their lightness and superior impermeability. In sultry latitudes, the last named is to be preferred to the second, from its being both waterproof and not adhering together so closely when one part touches another, that it becomes inseparable or cannot be drawn asunder without being torn and spoiled. A vast amount of gum elastic cloth is thus ruined. From its neatness and flexibility, however, it is an excellent article for mattress covers, counterpanes, capes, coats, cloaks and pantaloons in damp and mild climates, and may be very useful in the most sultry, when care is taken to keep apart the different folds and to prevent any portion of the side on which the gum is spread from touching any other, when the articles are laid by and not in use. Considering how much these waterproof clothes contribute to comfort and health, it is wonderful that they have not yet come into general use for sailors, and that none are provided by Government for them. They make much less use of gum-elastic shoes, which are so much worn on land, and so indispensable in keeping the feet dry. When gum-elastic overshoes cannot be got for this purpose, an excellent substitute will be found by wiping off all dirt and blacking from shoes or boots, and then several times smearing them with a composition of gum-elastic clipped to pieces, softened with pure spirits of turpentine and dissolved by boiling in whale oil or some other animal one. For the soles, several coats of copal varnish will answer the same purpose; and this is to be preferred, from its giving them greater firmness and durability.

Besides the ordinary articles of the materia medica to cure disease, we have to get a supply of others to prevent it, or those termed disinfecters. The most commonly used, and one of the most efficacious is chloride of lime, but it is objectionable from its great bulk and weight proportioned to the amount of chlorine it contains, and from its deliquescing when exposed to moisture. The other chlorides, as those of zinc and lead, are therefore preferable; but for instantaneous disengagement of chlorine I prefer the mixture of table salt, sulphuric acid and black oxide of manganese in due proportion—that is, having three parts of this to four of the first article and four of the second one, diluted in four parts of water. Sulphuric acid, also, poured on nitre, disengages a large amount of disinfecting gas or nitrous acid fumes, and constitutes the famous one of Dr. James Carmichael Smith, of Dublin. The proportion is a half fluid ounce of the acid to two drachms of the salt. The burning of sulphur and nitre together, the fumes of gunpowder, tar, tobacco and vinegar, and white washing with slacked lime or chloride of lime, may likewise be useful; and we may employ Labarraque's solution of chlorinated soda, which is certainly good to destroy the fœtor of sores and wounds. We also may use Sir William Burnett's solution of the chloride of zinc, and yet not have a healthy ship if she be not cleaned, particularly in the hold, where such accumulations of noxious substances collect. No disinfecting agent could be got to counteract their deleterious action on a crew, and all the patent air pumps which could be worked by them could never throw out of a ship thus loaded with filth all the poisonous gases generated in her during a long sojourn in a hot climate. Nor when small pox or any other truly contagious disease is prevalent on board, can we trust exclusively to any such means for disinfecting the air in a vessel, and rendering it innocuous, as well as inoffensive to our senses.

